# Heat shock response and ionstasis: axis against neurodegeneration

**DOI:** 10.18632/aging.100517

**Published:** 2012-12-18

**Authors:** Nikos Kourtis, Vassiliki Nikoletopoulou, Nektarios Tavernarakis

**Affiliations:** ^1^ Howard Hughes Medical Institute and Department of Pathology, New York University School of Medicine, New York, NY 10016, USA; ^2^ Institute of Molecular Biology and Biotechnology, Foundation for Research and Technology, Heraklion 71110, Crete, Greece

“*That which does not kill us, makes us stronger*” This famous quote by Friedrich Nietzsche is exemplified by the phenomenon of “hormesis”. Exposure of organisms to mild stress fortifies them against subsequent, more severe insults. Yet, the relevant molecular mechanisms remain poorly understood.

Although heat stroke is often fatal and survivors may suffer permanent neurologic damage, the mechanisms underlying heat stroke-induced cell and tissue injury remain elusive. To gain insight, we employed the nematode *Caenorhabditis elegans* to simulate hyperthermia [1]. We observed that heat stroke triggers pervasive necrotic cell death and neurodegeneration in *C. elegans*. Interestingly, preconditioning of animals at mildly elevated temperature protects against subsequent heat-induced damage. The heat shock transcription factor HSF-1 and the small heat shock protein (sHSP) HSP-16.1 mediate cytoprotection after preconditioning. Interestingly, overexpression of *hsp-16.1* suppresses necrosis, bypassing the need for *ab initio* activation of the heat shock response pathway. HSP-16.1 colocalizes with the Ca^2+^/Mn^2+^ ATPase PMR-1 in the Golgi. Notably, worms lacking PMR-1 do not benefit from preconditioning and remain susceptible to heat stroke damage. This indicates that the hormetic effect of preconditioning converges on Golgi and the PMR-1 Ca^2+^ pump. In addition, loss of *pmr-1* abolishes the protective effect of *hsp-16.1* overexpression, suggesting that PMR-1 is an important substrate of this sHSP. Interestingly, preconditioning also suppresses cell death inflicted by diverse insults. Notably, this protective mechanism is highly conserved. We found that murine neurons suffer necrosis upon heat stroke, while prior preconditioning prevents death.

sHSPs constitute a diverse family of proteins with multiple roles. Several ageing theories suggest that longevity positively correlates with the ability of the cell and the organism to resist stress. Ageing influences both general and organelle-specific stress response pathways [2]. Distinct experimental approaches have identified proteins that are abundant in long-lived worms [3]. Intriguingly, the most consistently represented subset is the sHSP group, including the HSP-16 family. We propose that HSP-16.1 mediates its protective effect partly by preserving cellular ionic homeostasis, which is perturbed in the stressful context of ageing. How could sHSPs protect under unfavorable conditions? In stressed cells, ATP levels drop significantly leading to fatal aggregation of damaged proteins. sHSPs protect proteins from thermal denaturation and irreversible aggregation in an ATP-independent manner. We propose that sHSPs constitute one of the cell's first lines of defense against cell death.

Is the Golgi an organelle to which different hormetic treatments converge? Ischemic/reperfusion brain injury (IRI) is a severe event characterized by ion homeostasis deregulation leading to necrosis. Ischemic preconditioning (IPC) involves a sub-lethal ischemia, which augments tolerance of the nervous system to subsequent, otherwise lethal ischemia. Previous studies showed that IRI causes a reduction of SPCA activity (the PMR-1 homolog) [4]. SPCA, similarly to other P-type ATPases, is selectively damaged during ischemia. However, preconditioning has a protective effect on SPCA activity. These results, together with ours, indicate that different types of preconditioning converge on protecting PMR-1 function and prevent deregulation of Ca^2+^ homeostasis. Interestingly, sHSP overexpression in transgenic animals and cultured cardiomyocytes, protects cells against necrosis following IRI [5].

Considering population genetics, some individuals are more sensitive to hyperthermia and suffer heat stroke under conditions where others demonstrate mild symptoms. Due to its amenability to genetic manipulations, *C. elegans* provides a valuable platform for the identification and characterization of genetic factors and polymorphisms that underlie altered responses to heat stroke. Heat stroke is a form of hyperthermia accompanied by activation of the heat shock response. Therefore, conditions that alter the expression levels of HSPs such as aging may aggravate the outcome of heat stroke. Interestingly, heat stroke incidents are more frequent among the elderly. Studies in *C. elegans* are well-poised to reveal aging-related alterations in stress responses that increase susceptibility to heat stroke. Despite the detrimental consequences of heat stroke, there is no pharmacological agent approved to combat this disease. Given that *C. elegans* provides an attractive model for high-throughput screens, our model may serve as screening platform for compounds that prevent extreme temperature-induced cytotoxicity.

Are components of organelle-mediated ionstasis targets of heat shock proteins? Increased expression of Hsp72 ameliorates the dystrophic pathology in mouse models of Duchenne muscular dystrophy [6]. While the sarco/endoplasmic reticulum Ca^2+^-ATPase (SERCA) is impaired in these mice, Hsp72 interacts with SERCA to preserve its function. These findings, combined with ours, indicate that organelle-specific Ca^2+^ channels and pumps might be important targets of heat shock proteins, and that these interactions preserve organelle ionstasis under stress. In addition to acute stress, also chronic stress appears to impact components of the organelle-mediated ionstasis machinery. In a murine chronic restrain stress model, the function of neuronal type 2 ryanodine receptor/calcium release channel (RyR2) is perturbed, resulting in intracellular Ca^2+^ leakage [7]. Inhibition of Ca^2+^ leakage prevents cognitive dysfunction.

Currently, drug discovery efforts are focused on blocking Ca^2+^ flow through membrane channels in pathological conditions. Our findings indicate that cells possess powerful ionstasis machinery, the components of which might serve as novel drug targets for multiple pathologies. It was recently shown that treatment of mice carrying a mutation in type I ryanodine receptor, that leads to death after short exposure to elevated temperature, with 5-aminoimidazole-4-carboxamide ribonucleoside (AICAR) prevents heat-induced death [8]. AICAR confers its protective effect by reducing Ca^2+^ leak from the sarcoplasmic reticulum to the sarcoplasm. Thus, harnessing endogenous, stress-protective mechanisms of the cell might lead to novel therapeutic approaches for a multitude of human pathologies.

**Figure 1:**
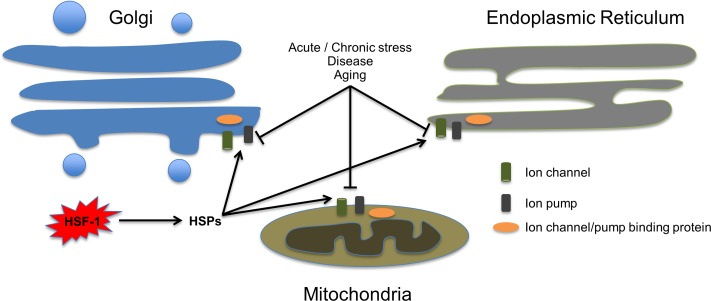
Heat shock proteins reinforce organelle-mediated ionstasis. Extracellular or intracellular stress impairs the function of organelle-specific ion channels, pumps and ion binding proteins (depicted as cylinder, rectangular and oval shapes respectively). Activation of HSF-1 upregulates heat shock protein levels, which in turn contribute to preserve intracellular ion-storage compartments, and prevent runaway perturbation of ionstasis and consequent cell death.

## References

[R1] Kourtis N, Nikoletopoulou V, Tavernarakis N (2012). Nature.

[R2] Kourtis N, Tavernarakis N (2011). EMBO J.

[R3] Murphy CT, McCarroll SA, Bargmann CI (2003). Nature.

[R4] Pavlikova M, Tatarkova Z, Sivonova M (2009). Cell Mol Neurobiol.

[R5] Hollander JM, Martin JL, Belke DD (2004). Circulation.

[R6] Gehrig SM, van der Poel C, Sayer TA (2012). Nature.

[R7] Liu X, Betzenhauser MJ, Reiken S (2012). Cell.

[R8] Lanner JT, Georgiou DK, Dagnino-Acosta A (2012). Nat Med.

